# Will mothers of sick children help their husbands to stop smoking after receiving a brief intervention from nurses? Secondary analysis of a randomised controlled trial

**DOI:** 10.1186/1471-2431-13-50

**Published:** 2013-04-08

**Authors:** Sophia S C Chan, David C N Wong, Tai-Hing Lam

**Affiliations:** 1 School of Nursing, University of Hong Kong, 4/F William M W Mong Block, 21 Sassoon Road, Pokfulam, Hong Kong; 2 School of Public Health, University of Hong Kong, 5/F William M W Mong Block, 21 Sassoon Road, Pokfulam, Hong Kong

**Keywords:** Mothers of sick children, Nurse-led education, Paediatric settings, Randomised controlled trial, Smoking husband

## Abstract

**Background:**

Second-hand smoke is a severe health hazard for children. Clinical guidelines suggest that nurses advise smoking parents to quit when they accompany their sick children to paediatric settings, but the guidelines did not mention what nurses can do if the parents are not with the children. This study examines the effectiveness of a low-intensity, nurse-led health instructional initiative for non-smoking mothers, to motivate them to take action to help their husbands stop smoking.

**Methods:**

This was a randomised controlled trial and 1,483 non-smoking women, who were living with husbands who do smoke, were recruited when they accompanied with their sick children on hospital admission in general paediatic wards/outpatient departments of four hospitals in Hong Kong. The women were randomly allocated into intervention and control groups. The former received brief health education counselling from nurses, a purpose-designed health education booklet, a “no smoking” sticker, and a telephone reminder one week later; the control group received usual care. The primary outcome was the women”s action to help their smoking husbands stop smoking at 3-, 6- and 12-month follow-ups.

**Results:**

A higher proportion of women in the intervention than the control group had taken action to help their husbands stop smoking at the 3-month (76% vs. 65%, P < .001), 6-month (66% vs. 49%, P < .001) and 12-month (52% vs. 40%, P < .001) follow-ups. Women who had received the intervention, had better knowledge of the health hazards of smoking, higher intention to take action, perceived their husbands’ willingness to stop/reduce smoking, had previously advised their husbands to give up smoking, were aware of their husbands’ history of smoking and, were aware that their husbands had made an earlier quit attempt and intended to help them stop smoking at the follow-ups.

**Conclusions:**

A brief health education intervention by nurses in paediatric settings can be effective in motivating the mothers of sick children to take action to help their husbands quit smoking. We recommend adding the following to the clinical practice guidelines on treating tobacco use and dependence: ‘Nurses should offer every non-smoking mother of a sick child brief advice to encourage their husbands to stop smoking’.

**Trial registration:**

Current Controlled Trials ISRCTN72290421.

## Background

This paper reports a secondary data analysis intended to test the effectiveness of brief counselling by nurses to encourage mothers of sick children to advise their husbands to stop smoking. The original study was a randomised controlled trial in general paediatric wards, and it showed that a brief counselling session by nurses given to non-smoking women was effective in changing the behaviour of smoking husbands, motivating women to take action to protect their children from second-hand household smoke exposure at 3-month follow-up [1,2]. While we demonstrated in the previous paper that it was possible for nurses to deliver the quitting message to the fathers of sick children through the non-smoking spouse, it remained unclear whether the brief intervention was effective in motivating women to help their husbands quit smoking. Furthermore, no study has examined the factors inducing women to influence their husbands in this way, although spousal support has long been discussed as a key factor in successful quitting [3-5].

Both active and passive tobacco smoke is a type–I carcinogen with immediate and direct hazards to health [6,7]. Parental smoking is a serious health hazard to the whole family, both to the smoking parents and to non-smoking family members who are exposed to second- and third-hand smoke in the household [8]. Quitting smoking is essential to reduce the health hazards of smokers and to remove the risk of SHS exposure to their family members. In the past 30 years, Hong Kong has made remarkable efforts in tobacco control, which have resulted in a progressive decrease in smoking prevalence from 23.3% in 1982 to 11.8% in 2008 [9]. The government first brought in a tobacco control ordinance in 1982, and subsequent amendments have been made to restrict tobacco advertising, sponsorship in sport and other entertainment areas by tobacco companies, cigarette sales to those below 18; action has also been taken against cigarette smuggling, and no-smoking areas have been extended to all indoor workplaces and public spaces, including restaurants. Alongside this success, however, there is the unintended consequence of possible displacement of smoking to Hong Kong homes. With smoke-free legislation operating in all indoor public areas (including the whole residential area under public estates and the whole building, apart from inside apartments), many parents have simply moved their smoking to their homes, and 14% of primary school children reported an increased exposure to household SHS in 2008 [10]. The problem could be severe in densely populated cities with crowded living quarters (in Hong Kong, the median living area per person is just 11.4 m^2^ in public estates), where smoking hygiene (smoke > 3 m away from non-smoking household members [8,11]) is hardly promoted [10,12]. This could increase the health threat to non-smoking women and children living with a smoker. As in most Asian cities, while women’s smoking prevalence is low (3% in Hong Kong [9]), they have little awareness about the health hazards of SHS exposure, and fewer than half non-smoking women would always advise their husbands to stop smoking [2,13].

The hospitalisation of sick children is an important juncture where nurses can teach women to protect their children from SHS [14]. To deal with the increasing threat of such exposure in the home, women should advise and assist their husbands to quit smoking, in order to avoid the health hazards of both active and passive smoking. Being at the frontier of patient care, nurses have a responsibility to provide smoking cessation counselling to smoking parents when they bring their children to hospital [15-17]. However, few nurses would assist smokers to quit in the clinical setting [18], because of heavy workload and the lack of time [19]. Furthermore, in paediatric wards, nurses rarely have the chance to meet smoking fathers directly, as it is usually the mother who takes care of the child on admission and during hospitalisation. To address this practical situation, the present paper describes the current practice of sick children’s mothers in advising and helping their husbands to stop smoking; reports the effectiveness of a low-intensity nurse-led intervention during a child’s hospitalisation in motivating their mothers to take action to help their husbands to quit; and examines factors predicting such action.

## Methods

### Study design

A multi-centred randomised controlled trial (RCT) was conducted in the general paediatric wards/outpatient departments of four hospitals in Hong Kong from November 1997 to September 1998. The primary purpose was to examine the impact of a low-intensity five-minute intervention (brief advice) delivered by nurses via non-smoking women to protect their children from household SHS exposure and help their husbands give up [2]. This paper examined whether the intervention might motivate the mothers of sick children to help their husbands in this way.

### Subjects

The study targeted women accompanying their sick children on admission to hospital. The inclusion criteria covered (a) non-smoking women bringing their sick children to the paediatric wards or outpatient departments; (b) fathers being current smokers; (c) mother, father and child living together in the same household; and (d) women able to communicate in Cantonese/Chinese. Cases where children were admitted to hospital with relatives other than the mothers or with maids were excluded from the study. A total of 1,483 women were recruited and randomly allocated into intervention (n = 752) and control (n = 731) groups.

The family characteristics of the two groups were comparable: women aged 34.4 years (SD = 6.5) on average, the majority living in Hong Kong for over 10 years (64%), most having secondary education (71%); and nearly two thirds housewives (66%). The mean age of fathers was 39.1 years (SD = 7.3), nearly two thirds had secondary education (67%) and most were in blue-collar occupations (81%). The mean age of the children was 4.8 years (SD = 4.3) and 68% were male, the major medical complaints included respiratory symptoms (50%), fever (48%) and gastro-intestinal symptoms (19%), and the majority had been admitted to hospital before (60%). Slightly over half the families lived in privately owned or rented housing (56%) and nearly three quarters (74%) had a family income of less than HK$20,000 per month. (The median monthly domestic household income was HK$18,705 during the survey period; US$1 = HK$7.8). Most families suffered from household SHS exposure (85%). Detailed characteristics have been reported previously [2,14].

### Procedures

**Figure 1:**
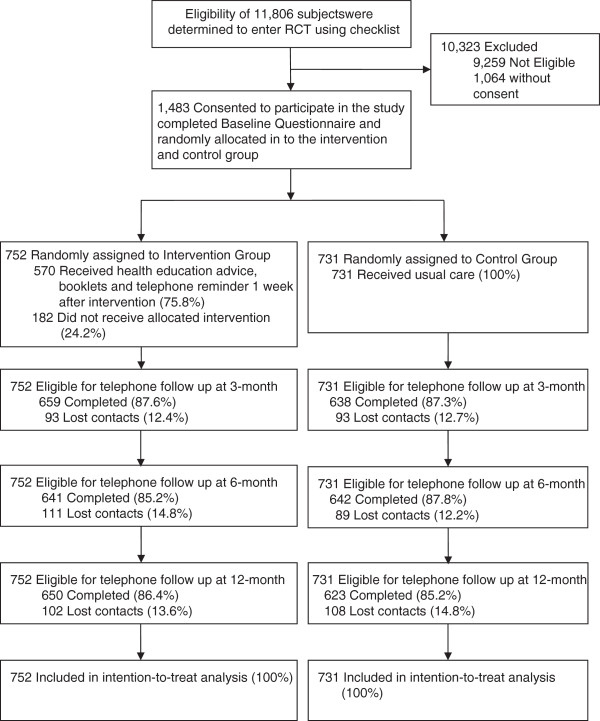
Flow chart of study participants through the trial.

Figure 1 illustrates a flow chart of participants recruited to the RCT. Trained nurses first screened the eligibility of the child’s family, by means of a checklist, when the sick child was first admitted. A total of 11,806 families were screened and 2,524 met the inclusion criteria. Eligible women were invited to participate, 1,483 giving written consent and completing a self-administered baseline questionnaire. Subjects were then randomly allocated into intervention (n = 752) and control (n = 731) groups by means of computer-generated random numbers, and the allocation results were sealed in serial-numbered opaque envelopes. Only nurse counsellors and the research assistant knew the results of the allocation, as they were responsible for enrolling and assigning subjects to groups and administering the intervention, but not the data analyst or project investigators. Participants were also blinded to the allocation, as they were not told which group they were in and did not know the intervention details of the alternative group. After randomisation, the intervention group received a brief session of health education counselling from nurses, while the control group received no more than the usual care. Both groups received a 10-minute telephone follow-up by trained nurse interviewers at 3, 6, and 12 months to assess the outcomes. The retention rate was 88%, 87% and 86% respectively, and was similar for both groups. No significant difference was found in baseline characteristics between respondents and non-respondents. A pilot study of 75 non-smoking women had been conducted before the implementation of the main study to test study procedures and feasibility.

The instruments included the baseline questionnaire (BQ) and subsequent questionnaires at 3, 6 and 12-month follow-ups. The BQ was designed based on a conceptual framework with a comprehensive literature review, assessing the smoking behaviour of sick children’s fathers and their quitting history, the children’s SHS exposure in the household, the women’s knowledge of and attitudes to active and passive smoking, any action to protect their children from SHS exposure or to help their husbands to stop smoking, and demographic data. The follow-up questionnaires, based on the BQ, assessed the smoking status of sick children’s fathers, mothers’ action (1) to protect their children from SHS exposure and (2) to help their husbands give up, and the children’s health status [17]. Both baseline and follow-up questionnaires were pre-evaluated for face and content validity by a panel of local and international tobacco-control experts (levels of agreement ranged from 0.73 to 0.94), and pre-piloted by a group of University of Hong Kong post-graduate students to ensure the instruments were both comprehensive and feasible for the study. The reliability of the instruments was also tested. A total of 100 women were randomly selected to receive further calls one week after they had completed the 3-month telephone follow-up survey. They were invited to answer nine questions extracted from the original 3-month follow-up questionnaire on: husband’s smoking and quitting behaviour, children’s exposure to SHS, action taken to protect children from SHS exposure, and action to advise and assist husbands to stop smoking. The level of agreement ranged from 85% to 100% on the above items (Kappa statistics ranged from 0.65 to 1).

### Intervention

The intervention study was conceptualized based on the transtheoretical model [20] and theory of planned behaviour [21] to motivate women to help their husbands to quit. The intervention was designed to raise women’s awareness of the health hazards of passive smoking (to increase behavioural beliefs), to strengthen their need to take action (to increase normative beliefs) and to strengthen their capacity (to increase their self-efficacy). The intervention group received a nurse-led intervention which included (1) a brief (five-minute) standardised counselling session about the impact of SHS on their sick children, and preventive measures and advisory techniques aimed at motivating their husbands to stop smoking, such as asking them to read a self-help booklet on smoking cessation provided by the nurse counsellor (Additional file 1: Appendix 1); (2) a purpose-designed health education booklet for women, *Protect Yourself and Your Children: What to Do About Second-Hand Tobacco Smoke*, and another self-help guide *Smart Move: a Stop Smoking guide* for; (3) a ‘No smoking’ sign for women to put up in their home and remind their husbands not to smoke; and (4) a telephone reminder one week later. Since this was a brief intervention, the contents were limited and women were not asked to take any further action for relapse prevention if their husbands had indeed stopped smoking. Furthermore, our intervention did not emphasize on encouraging their husbands to smoke away from their children since our main focus was to encourage the husbands to quit smoking. No intervention, nothing beyond standard care, was given to the control group. The details of the intervention have been published previously [2,14]. The intervention has been pilot-tested to ensure it can be conducted in busy hospital settings. No financial incentives were given to the participants throughout the study.

### Measures

The primary outcome was whether women had taken action to help their husbands quit smoking at 3-, 6- and 12-month follow-ups (Yes/No). Any of the following actions would count: (1) helping husbands to set a date to quit; (2) putting up a ‘No Smoking’ sign at home; (3) asking husbands to read a health education booklet on giving up; (4) advising them to seek help from healthcare professionals; (5) reminding them that, by stopping smoking, their children would be healthier and less likely to become smokers in the future; and (6) discussing the matter with them and understanding their needs in the process of quitting. We counted any action taken by the women since the last follow-up, unless their husbands had actually stopped smoking. The secondary outcome was whether women had given direct advice to their husbands on quitting smoking at 3-, 6- and 12-month follow-ups (Yes/No).

The key predicting variable was the intervention effect (intervention vs. control). Additional independent variables at baseline were examined: (1) women’s perceptions, including their intention to help husbands quit smoking in the next month (Y/N), and perceiving they would quit or reduce smoking in the next three months (Y/N); (2) women’s knowledge of the health hazards of active and passive smoking (a scale of 0–5, Cronbach’s alpha = 0.80); (3) women perceiving that smokers can quit successfully if they are determined (Y/N); (4) women perceiving that family support and encouragement can help a smoker quit (Y/N); (5) women’s prior actions to advise their husbands to quit smoking (Y/N) and any practical action taken to help them to give up (Y/N); and (6) fathers’ smoking history (> 10 years, 6–10 years or ≤ 5 years), any attempt to stop in the past 12 months (Y/N) and if so whether they had stopped for seven days or longer (Y/N). Women’s proxy reports on their husbands smoking status (stopped/not stopped) at 3-, 6- and 12-month follow-ups were also included.

### Data analysis

Data were analysed using SPSS 18.0 (SPSS Inc, Chicago, IL). Chi-square statistics and independent two-sample t-tests were used to compare the baseline profiles of women in the intervention and control groups, and their current practice in advising and helping their husbands to quit smoking. Odds ratios were used to test the effectiveness of the intervention in increasing the likelihood of women to advise and take action to help their husbands quit smoking. We excluded women from the analysis who reported that their husbands had already given up smoking, since actions such as setting a date for quitting, would not be applicable in that context. To examine the factors predicting women’s actions in helping their husbands quit smoking, we applied a generalised estimating equation (GEE) in an unstructured correlation matrix with binary logistic link function in the analysis. All the predicting variables were free of multi-collinearity problems (tolerance values ranged from .70 to .98). The interaction effects across the predicting variables were insignificant and are hence excluded. A ‘carry-forward’ approach was applied in intention-to-treat analysis to handle missing responses at 3-, 6- and 12-month follow-ups [22]. All statistical analyses were tested using a 5% level of significance.

### Ethical considerations

The study has complied with the Helsinki Declaration (http://www.wma.net/en/30publications/10policies/b3/index.html). It was approved by the Faculty of Medicine Ethics Committee of our institute in September 1997 (EC-150), and was registered in the Current Controlled Trials: ISRCTN72290421. Before the study began, full details were given to all participants, who were assured of confidentiality and gave their informed written consent. All data were reported in a collective fashion, and participants could withdraw at any time without affecting their children’s treatment.

## Results

### Current practice of sick children’s mothers in advising and helping their husbands to quit smoking, at baseline

At baseline, mothers of sick children had some knowledge of the health hazards of active and passive smoking (mean = 3.83, SD = 1.52, range = 0–5). Around two thirds believed that smokers could quit successfully if they were determined (69%), and that family support and encouragement could help them to do so (66%). Most intended to help their husbands to quit in the next month (84%), and over half thought they would stop or reduce smoking in the next three months (56%). Most reported they had at some time advised their husbands to quit (90%), but fewer than half had ever taken specific action to help them do so (44%). Slightly less than a third of women had ever urged their husbands to stop smoking so that their children would be healthier and less likely to become smokers in the future (31%). Very few had ever suggested that their husbands might undergo smoking cessation counselling (1.6%). No significant difference was found between the intervention and control groups, except that a slightly higher proportion of women in the former group had at some point asked their husbands to read a self-help smoking cessation booklet (P = .04) (Table 1).

**Table 1:** Mothers’ knowledge, attitudes, perceptions and actions taken, at baseline (n = 1483)

	**Intervention (n = 752)**	**Control (n = 731)**	
**Baseline profile**	**n (%†)**	**n (%†)**	**P-value**
*Knowledge*			
- Health hazards of active and passive smoking (0 – 5)	Mean = 3.81	Mean = 3.85	.67
	SD = 1.51	SD = 1.52	
*Attitudes*			
- ‘Smokers can quit successfully if they are determined”’	533 (70.9)	488 (66.8)	.09
- ‘Family support and encouragement can help a smoker quit’	501 (66.6)	475 (65.0)	.51
*Perceptions*			
- Intend to help their husbands to quit smoking in the next month	619 (85.1)	580 (81.8)	.09
- Think their husbands will quit or reduce smoking in the next three months	414 (56.4)	387 (54.8)	.54
*Advice*			
- Have at some time advised their husbands to quit smoking	663 (90.2)	635 (88.7)	.35
*Actions*			
- Have at some time urged their husbands to stop smoking, so that their children will be healthier and less likely to become smokers in the future	234 (32.1)	217 (30.5)	.53
- Have at some time asked their husbands to read a self-help smoking cessation booklet	81 (11.1)	56 (7.9)	.04
- Have at some time talked to their husbands about understanding their needs in quitting	72 (9.9)	50 (7.0)	.06
- Have at some time put up a ‘No Smoking’ sign at home	53 (7.3)	51 (7.2)	.99
- Have at some time helped their husbands to set a date for giving up	36 (4.9)	21 (3.0)	.06
- Have at some time asked their husbands to undergo smoking cessation counselling	12 (1.6)	11 (1.5)	.99
- *Have at some timer taken any of the above actions to help their husbands to stop smoking*	*334 (45.8)*	*295 (41.5)*	*.10*

† Excluding missing responses.

### Effectiveness of nurse-led intervention in motivating women to advise and help their husbands to quit smoking

Excluding women who reported that their husbands had quit smoking, a higher proportion of the intervention group had advised their husbands to give up at 3-month (OR = 1.46, 95% CI = 1.17 – 1.80), 6-month (OR = 1.23, 95% CI = 1.00 – 1.53) and 12-month (OR = 1.09, 95% CI = 0.88 – 1.35) follow-ups, although the difference became statistically insignificant at 12-month. Similarly, more women in the intervention group took at least one action to help their husbands quit smoking at 3-month (OR = 1.69, 95% CI = 1.34 – 2.14), 6-month (OR = 2.00, 95% CI = 1.61 – 2.50) and 12-month (OR = 1.62, 95% CI = 1.30 – 2.01) follow-ups (Table 2). During the 3-month follow-up, over half of the women in the intervention group reminded their husbands the importance of quitting to the health of their children and reducing the chance of their children becoming smokers in the future (66%), and asked them to read a self-help smoking cessation booklet (52%). Fewer women in the intervention group put up a ‘No-smoking’ sign at home (44%) or talked to their husbands about understanding their needs during the quitting process (35%). Very few women in the intervention group encouraged their husbands to seek help from healthcare professionals (5.7%) or helped their husbands to set a date for quitting (4.3%). Nevertheless, the proportion of women who took the above actions was higher in the intervention than in the control group (Additional file 1: Appendix 2).

**Table 2:** Mothers’ actions to help their husbands to quit smoking, at 3-, 6- and 12-month follow-ups (n = 1,483¶)

**(%)**	**Intervention**	**Control**	**OR (95% CI)**
Have advised their husbands to quit smoking			
• 3-month†	441/696 (63.4%)	378/696 (54.3%)	1.46 (1.17, 1.80)***
• 6-month‡	354/682 (51.9%)	313/671 (46.6%)	1.23 (1.00, 1.53)*
• 12-month‡	312/667 (46.8%)	296/663 (44.6%)	1.09 (0.88, 1.35)
Have taken action(s) to help their husbands to quit smoking §			
• 3-month†	527/696 (75.7%)	451/696 (64.8%)	1.69 (1.34, 2.14)***
• 6-month‡	650/682 (66.0%)	330/671 (49.2%)	2.00 (1.61, 2.50)***
• 12-month‡	349/667 (52.3%)	268/663 (40.4%)	1.62 (1.30, 2.01)***

Notes: * p < 0.05; ** p < 0.01; *** p < 0.001.

† Mothers who did not take part in any follow-ups were assumed not to have taken any action to help their husbands to give up.

‡ Mothers who only responded one or two follow-ups were assumed to have the same responses as those of the previous follow-up (‘carry-forward method’).

§ Any of the following actions counted: (1) helping husbands to set a date for giving up; (2) putting up a ‘No Smoking’ sign at home; (3) asking their husbands to read a quit-smoking health education booklet tailor-made for this study; (4) advising them to seek help from healthcare professionals; (5) encouraging them to stop smoking so that their children will be healthier and less likely to become smokers in the future; and (6) discussing matters with them to understand their needs during the quitting process.

¶ Excluding mothers who reported their husbands had already stopped smoking. Proxy reports of fathers’ smoking status: at 3-month follow-up (91 stopped, 1,392 still smoking); at 6-month follow-up (130 stopped, 1,353 still smoking); at 12-month follow up (153 stopped, 1,330 still smoking).

In general, the proportion of women who helped their husbands to quit smoking decreased over time. Still, 52% women in the intervention group took at least one action to help their husbands stop smoking at 12-month follow-up (among families where the husbands were still smoking). Among those who reported that their husbands had quit, 31% of the intervention group took at least one action to help their husbands stop smoking at 12-month follow-up (Table 2).

### Predictors of women’s action to help their husbands to stop smoking

Table 3 shows findings based on a generalised estimating equation (GEE). The nurse-delivered intervention could significantly increase the likelihood of women taking action to help their husbands stop smoking (Adj. OR = 1.72, 95% CI = 1.46 – 2.02). Also predictive of women’s actions at follow-ups were better knowledge of the health hazards of smoking (Adj. OR per unit increase = 1.07, 95% CI = 1.01 – 1.13), an intention to take action, perceptions of their husbands’ willingness to quit or reduce smoking, and having previously advised or taken action to help their husbands quit smoking (Adj. OR ranged from 1.21 to 1.51, P-values ranged from < .001 to .03). A husband’s longer smoking history over 10 years (Adj. OR = 1.90, 95% CI = 1.49, 2.42) or over 5 years (Adj. OR = 1.79, 95% CI = 1.33, 2.40), and his attempt at stopping in the past 12 months (Adj. OR = 1.28, 95% CI = 1.01, 1.60) predicted his wife helping him to quit. Women’s attitudes such as ‘smokers can quit successfully if they are determined’ did not significantly predict their actions at follow-ups. These findings were adjusted for husbands’ smoking status during follow-ups (Table 3).

**Table 3:** Generalised estimating equation model of mothers taking any action to help their husbands stop smoking at 3-, 6- and 12-month follow-ups (n = 1283)†

**Predictors**	**Adj. OR‡**	**95% CI**	**P-value**
Treatment group			
- Intervention	1.72	(1.46, 2.02)	<.001***
- Control	1		
*Baseline factors (mothers’ knowledge)*			
• Knowledge of the health hazards of active and passive smoking (scale: 0 – 5)	1.07§	(1.01, 1.13)	.03*
*Baseline factors (mothers’ attitudes)*			
• Perceiving smokers can quit successfully if they are determined	1.05	(0.87, 1.27)	.62
• Perceiving that family support and encouragement can help a smoker quit	0.94	(0.77, 1.14)	.53
*Baseline factors (mothers’ perceptions)*			
• Intend to help their husbands quit smoking in the next month	1.51	(1.21, 1.88)	<.001***
• Think their husbands will quit or/reduce smoking in the next three months	1.29	(1.07, 1.56)	<.01**
*Baseline factors (mothers’ actions)*			
• Have at some time advised their husbands to quit smoking	1.47	(1.11, 1.94)	<.01**
• Have at some time taken action(s) to help their husbands to quit smoking	1.21	(1.02, 1.44)	.03*
*Baseline factors (fathers’ smoking and quitting profiles)*			
• Fathers’ smoking history			
- > 10 years	1.90	(1.49, 2.42)	<.001***
- 6 to 10 years	1.79	(1.33, 2.40)	<.001***
- ≤ 5 years	1		
• Fathers making an attempt to stop in the past 12 months	1.28	(1.01, 1.60)	.04*
• Fathers stopping smoking for seven days or longer in that attempt	1.07	(0.89, 1.29)	.49

Notes: OR denotes odds ratio; CI denotes confidence interval; * p < 0.05; ** p < 0.01; *** p < 0.001.

* p < 0.05; ** p < 0.01; *** p < 0.001.

† The model excludes 200 cases with incomplete baseline data.

‡ Adjusted for fathers’ smoking status at follow-up and all other variables in the model.

§ Adj. OR per unit increase.

## Discussion

Our study shows that most women are willing to help their husbands stop smoking, especially when they perceived their husbands are willing to do so. It also shows that a simple low-intensity (five-minute) intervention by nurses could be effective in encouraging women to take action and assist their husbands to quit. Although the intervention did not have a very large effect, the findings could be important in Asian regions such as Mainland China, where smoking rate among male is very high (59.5%) but very low among females (3.7%). The literature consistently reports a dilemma for women in a Chinese society between health concerns about smoking and the traditional need to be supportive and maintain family harmony, which could have discouraged them from advising their husbands to stop or reduce their smoking [14,23,24]. The nurse-led intervention could be valuable in empowering women to go beyond that dilemma, by providing expert opinion to increase women’s awareness of the adverse health effects on the family of the husband’s smoking habit; and by offering tips and suggestions for women’s action that might help their husbands quit smoking. This implies the possibility of extending the nurse’s role in promoting smoking cessation in an indirect way, through a non-smoking spouse. In particular, it is highly feasible for nurses to deliver brief advice to mothers of sick children in a busy paediatric ward/department, when they do not have the opportunity to meet the smoking fathers.

Our findings record a decrease in the intervention’s impact over time on women advising and helping their husbands to quit smoking. While the hospitalisation of sick children might have triggered non-smoking mothers to take action, the brief one-off intervention by nurses may not be sustainable when the children recover. Also, women’s advice and action may not be sustained if they do not receive positive feedback from their husbands. Some booster interventions, e.g. a short telephone follow-up, may be required to reinforce in women the notion that smoking is intolerable and that quitting the habit is urgent, as even smoking for a short period or at a low level can damage the health of all concerned [25,26].

Nearly a quarter of the women in the intervention group were reluctant to take any action to help their husbands quit smoking at 3-month follow up. Apart from their knowledge of the health hazards of SHS exposure and their previous experience in taking action, our findings showed that women’s perceptions also predict their actions. Some women may only have a weak intention to take action even though they have negative feelings toward their husbands’ smoking. Also, very few women encouraged their husbands to seek help to receive smoking cessation counselling. Possible reasons include avoidance of conflict and maintaining family harmony. However, concern of children’s health could motivate some husbands to be more receptive to the wives’ advice. Further studies are warranted on encouraging women to advise their husbands to refrain from smoking at home or near the children as an alternative, if they are not interested in quitting.*”*

### Limitations

There were several limitations to the study. First, about 17% (n = 182) of women were unable to receive the intervention at all because of the nurses’ heavy workload and the rapid patient turnover rate in paediatric units. By using an ‘intention to treat’ analysis, the intervention effect might have been underestimated. However, given the relatively high retention rate in this study (86% – 88% during 3, 6, and 12-month follow ups), the findings did not change much when we excluded all missing cases and re-run the analyses (data not shown here). Second, the open ward setting provided an inevitable opportunity for women in the intervention and control groups to communicate with each other, and it is possible that some women in the latter group might have been exposed to part of the intervention through this way. Finally, there is a possibility that some women, in either group, might have given socially desirable responses in the telephone follow-ups, since they were aware of being studied.

## Conclusion

Our secondary data analyses have illustrated the important role of nurses in instructing and empowering non-smoking women, in a paediatric hospital setting, who might not otherwise have taken any action to help their husbands quit smoking. The nurses’ brief intervention has huge potential benefits especially in regions with high male smoking prevalence and low female smoking prevalence, so as to capitalize the capacity of non-smoking women in tobacco control advocacy. We recommend adding to the clinical practice guidelines on treating tobacco use and dependence the following: ‘nurses should offer every non-smoking mother of a sick child brief advice to encourage their husbands stop smoking’.

## Competing interests

The authors declare that they have no competing interests.

## Authors’ contributions

CSSC and LTH wrote the proposal and C conducted the study and inspired the direction in writing this manuscript. WDCN reviewed the literature, carried out all data analyses and drafted the manuscript under C supervision. L further supervised Wong in critically revising the manuscript. All authors have approved the final version.

## Pre-publication history

The pre-publication history for this paper can be accessed here:

http://www.biomedcentral.com/1471-2431/13/50/prepub

## Supplementary Material

Additional file 1 **Appendix 1. **Contents of a brief (5-minute) health education counselling session from a nurse. **Appendix 2. **Women’s actions to help their husbands quit smoking at 3-, 6- and 12-month follow-ups (n=1,483). Click here for file
