# Supplementary material for: Will mothers of sick children help their husbands to stop smoking after receiving a brief intervention from nurses? Secondary analysis of a randomised controlled trial
Source: BMC Pediatr. 2013 Apr 8;13:50. doi: 10.1186/1471-2431-13-50 (PMC3626671; doi:10.1186/1471-2431-13-50)
Supplement: Additional file 1 — Appendix 1. Contents of a brief (5-minute) health education counselling session from a nurse. Appendix 2. Women’s actions to help their husbands quit smoking at 3-, 6- and 12-month follow-ups (n=1,483). [file 1471-2431-13-50-S1.pdf]

**Appendix 1** Contents of a brief (5-minute) health education counselling session from a nurse

1. Nurse greets the child's mother
2. Asks the following questions to start the conversation:
   - 'Does your husband smoke?' ☐ Yes ☐ No
   - If Yes, 'How many cigarettes does he smoke each day?' ___________
   - 'Does he smoke at home?' ☐ Yes ☐ No
   - "Does he ever consider giving up?' ☐ Yes ☐ No ☐ Don't know
3. Explain the following points:
   - Secondhand smoke (SHS) is the smoke in the air that comes from a burning cigarette or from the exhalation of a person smoking. If you live with a smoker, you become a passive smoker yourself, posing risks to your own and your child's health.
   - SHS is known to cause lung cancer, heart disease and other respiratory diseases in adult non-smokers. Children exposed to SHS at home are more likely to suffer from coughing, phlegm, wheezing, respiratory infections, middle-ear disease and asthma.
   - Here are some suggestions for protecting you and your child from second-hand smoke:
     - Move away from people if they are smoking
     - Open the windows when anyone is smoking
     - Ask anyone who smokes not to do so around you
     - Request that the cigarette be extinguished before it gets close to you or your children
     - Put up a 'No Smoking' sign at home
   - The best way to protect yourself and your children is to encourage the child's father to **quit smoking**. Please read this '**Protect Yourself and Your Children: What to Do About Second-Hand Tobacco Smoke'** health education booklet. Some good suggestions to help smokers quit:
     - Make it clear that you are concerned about their health, your own health and your child's health
     - Help them set a date for quitting
     - Remove all ashtrays from the house or office
     - Tell them their children will not only be healthier but also less likely to become smokers
     - Say you are glad they don't smoke anymore. Praise and encourage them every day for not smoking
     - Ask them what else you could do for them that would be helpful
   - Here is another health education booklet: 'Smart Move: A Stop-Smoking Guide' for the child's father to read. Actually, the support and encouragement of family members given to a smoker during the quit process is very important. You have a vital role in helping your husband quit. Helping him to set a specific date for doing so is a good starting point.
   - If you need more information about quitting, you can call the Department of Health 24-hour hotline, 2833 0011'
4. Do you have any questions?
5. After offering the above health advice, make sure the mother understands the message and remind her to read the health education booklets.

**Appendix 2** Women's actions to help their husbands quit smoking at 3-, 6- and 12-month follow-ups (n=1,483)

| Women's actions (%) | Intervention | Control | OR (95%CI) |
|---|---|---|---|
| Reminding their husbands that if they stop smoking their children will be healthier and less likely to become smokers in the future | | | |
| • 3-month† | 461/696 (66.2%) | 414/696 (59.5%) | 1.34 (1.07, 1.66)** |
| • 6-month‡ | 374/682 (54.8%) | 299/671 (44.6%) | 1.51 (1.22, 1.87)*** |
| • 12-month‡ | 309/667 (46.3%) | 257/663 (38.8%) | 1.36 (1.10, 1.70)** |
| Asking their husbands to read a self-help smoking cessation booklet | | | |
| • 3-month† | 360/696 (51.7%) | 154/696 (22.1%) | 3.77 (2.99, 4.76)*** |
| • 6-month‡ | 239/682 (35.0%) | 100/671 (14.9%) | 3.08 (2.37, 4.01)*** |
| • 12-month‡ | 96/667 (14.4%) | 34/663 (5.1%) | 3.11 (2.07, 4.67)*** |
| Talking to their husbands about understanding their needs in the quitting process | | | |
| • 3-month† | 245/696 (35.2%) | 214/696 (30.7%) | 1.22 (0.98, 1.53) |
| • 6-month‡ | 94/682 (13.8%) | 81/671 (12.1%) | 1.16 (0.85, 1.60) |
| • 12-month‡ | 30/667 (4.5%) | 33/663 (5.0%) | 0.90 (0.54, 1.49) |
| Putting up a 'No Smoking' sign at home | | | |
| • 3-month† | 309/696 (44.4%) | 68/696 (9.8%) | 7.37 (5.51, 9.87)*** |
| • 6-month‡ | 234/682 (34.3%) | 56/671 (8.3%) | 5.74 (4.18, 7.87)*** |
| • 12-month‡ | 210/667 (31.5%) | 62/663 (9.4%) | 4.45 (3.27, 6.06)*** |
| Helping their husbands to set a date for giving up | | | |
| • 3-month† | 30/696 (4.3%) | 17/696 (2.4%) | 1.80 (0.98, 3.29) |
| • 6-month‡ | 19/682 (2.8%) | 17/671 (2.5%) | 1.10 (0.57, 2.14) |
| • 12-month‡ | 2/667 (0.3%) | 3/663 (0.5%) | ¶ |
| Encouraging their husbands to seek help from healthcare professionals | | | |
| • 3-month† | 40/696 (5.7%) | 24/696 (3.4%) | 1.71 (1.02, 2.86)* |
| • 6-month‡ | 42/682 (6.2%) | 22/671 (3.3%) | 1.94 (1.14, 3.28)* |
| • 12-month‡ | 6/667 (0.9%) | 3/663 (0.5%) | ¶ |

Notes: OR denotes odds ratio; CI denotes confidence interval; * $p < 0.05$; ** $p < 0.01$; *** $p < 0.001$

† Women not taking part in any follow-ups were assumed not to have taken any action to help their husbands to quit smoking

‡ Women only responding to one or two follow-ups were assumed to have the same responses as those of the previous follow-up ('carry-forward method')

§ Excluding women who reported that their husbands had already quit smoking. Proxy reports of fathers' smoking status: at 3-month follow-up (91 stopped, 1,392 still smoking); at 6-month follow up (130 stopped, 1,353 still smoking); at 12-month follow up (153 stopped, 1,330 still smoking).

¶ The cell is smaller than 5.
